# Plasma proteins present in osteoarthritic synovial fluid can stimulate cytokine production via Toll-like receptor 4

**DOI:** 10.1186/ar3555

**Published:** 2012-01-08

**Authors:** Dong Hyun Sohn, Jeremy Sokolove, Orr Sharpe, Jennifer C Erhart, Piyanka E Chandra, Lauren J Lahey, Tamsin M Lindstrom, Inyong Hwang, Katherine A Boyer, Thomas P Andriacchi, William H Robinson

**Affiliations:** 1 GRECC, VA Palo Alto Health Care System, 3801 Miranda Ave., Palo Alto, CA 94304, USA; 2 Division of Immunology and Rheumatology, Department of Medicine, Stanford University School of Medicine, Stanford, CA 94305, USA; 3 Bone and Joint Center, VA Palo Alto Health Care System, 3801 Miranda Ave., Palo Alto, CA 94304, USA; 4 Department of Mechanical Engineering, Stanford University, Stanford, CA 94305, USA

## Abstract

**Introduction:**

Osteoarthritis (OA) is a degenerative disease characterized by cartilage breakdown in the synovial joints. The presence of low-grade inflammation in OA joints is receiving increasing attention, with synovitis shown to be present even in the early stages of the disease. How the synovial inflammation arises is unclear, but proteins in the synovial fluid of affected joints could conceivably contribute. We therefore surveyed the proteins present in OA synovial fluid and assessed their immunostimulatory properties.

**Methods:**

We used mass spectrometry to survey the proteins present in the synovial fluid of patients with knee OA. We used a multiplex bead-based immunoassay to measure levels of inflammatory cytokines in serum and synovial fluid from patients with knee OA and from patients with rheumatoid arthritis (RA), as well as in sera from healthy individuals. Significant differences in cytokine levels between groups were determined by significance analysis of microarrays, and relations were determined by unsupervised hierarchic clustering. To assess the immunostimulatory properties of a subset of the identified proteins, we tested the proteins' ability to induce the production of inflammatory cytokines by macrophages. For proteins found to be stimulatory, the macrophage stimulation assays were repeated by using Toll-like receptor 4 (TLR4)-deficient macrophages.

**Results:**

We identified 108 proteins in OA synovial fluid, including plasma proteins, serine protease inhibitors, proteins indicative of cartilage turnover, and proteins involved in inflammation and immunity. Multiplex cytokine analysis revealed that levels of several inflammatory cytokines were significantly higher in OA sera than in normal sera, and levels of inflammatory cytokines in synovial fluid and serum were, as expected, higher in RA samples than in OA samples. As much as 36% of the proteins identified in OA synovial fluid were plasma proteins. Testing a subset of these plasma proteins in macrophage stimulation assays, we found that Gc-globulin, α_1_-microglobulin, and α_2_-macroglobulin can signal via TLR4 to induce macrophage production of inflammatory cytokines implicated in OA.

**Conclusions:**

Our findings suggest that plasma proteins present in OA synovial fluid, whether through exudation from plasma or production by synovial tissues, could contribute to low-grade inflammation in OA by functioning as so-called damage-associated molecular patterns in the synovial joint.

## Introduction

Osteoarthritis (OA) is a degenerative disease of the joints that is characterized by destruction of articular cartilage, inflammation of the synovial membrane (synovitis), and remodeling of periarticular bone. Which of these pathogenic processes occurs first is unknown. One proposed scenario is that cartilage breakdown (due to injury or mechanical stress) releases components of the damaged extracellular matrix (ECM) into synovial fluid, and that these ECM components elicit the local production of inflammatory molecules by binding to receptors on resident synovial cells or infiltrating inflammatory cells [1,2]. The inflammatory molecules produced may in turn stimulate production of cartilage-degrading enzymes and recruit inflammatory cells to the affected joint [3,4], thus establishing a vicious cycle of cartilage destruction and inflammation that perpetuates and promotes the OA pathology. Therefore, OA has been described as a chronic wound in which molecules in synovial fluid function as damage-associated molecular patterns (DAMPs; that is, endogenous molecules produced during injury that signal through inflammatory toll-like receptors (TLRs) to effect tissue remodeling) [2,5,6]. Although the identities of the endogenous molecules that mediate synovial inflammation have yet to be confirmed in OA patients or animal models, a continuous supply of DAMPs could perpetuate the early response to injury and thereby damage the joint.

Besides ECM components, many other molecules may act as DAMPs [2]. One such molecule is fibrinogen, which stimulates macrophage production of chemokines in a TLR4-dependent manner [7-9]. Fibrinogen is present at abnormally high levels in OA synovial fluid [10], and the amount of fibrin (the thrombin-cleaved form of fibrinogen [11]) deposited in the synovial membrane correlates with the severity of OA [12]. Although classically a plasma protein, fibrinogen exudes from the vasculature at sites of inflammation, such as the inflamed OA joint, owing to the retraction of inflamed endothelial cells [11]. Fibrinogen is not the only protein to extravasate at sites of inflammation, however, and several other plasma proteins have been detected in OA synovial fluid [10,13]. The extravascular function of most of these plasma proteins is unclear. It is possible that, like fibrinogen, some of these plasma proteins could have an immunoregulatory role at sites of inflammation or tissue damage.

Inflammation is present even in the early stages of OA [14,15], and clinical signs of synovitis correlate with radiographic progression of knee OA [16]. Insight into the cause of synovial inflammation is therefore important in understanding the pathogenesis of OA. Here we used proteomic techniques to survey the proteins present in OA synovial fluid and to evaluate levels of inflammatory cytokines in OA serum and synovial fluid. We then determined whether a subset of the identified proteins could promote inflammation by functioning as immunostimulatory DAMPs.

## Material and methods

### Synovial fluid and serum samples

Serum and synovial fluid samples were obtained from patients with knee OA, patients with rheumatoid arthritis (RA), or healthy individuals under protocols approved by the Stanford University Institutional Review Board and with the patients' informed consent. Synovial fluid aspiration was performed by a board-certified rheumatologist by fine-needle arthrotomy, and the synovial fluid samples obtained were free from obvious contamination with blood or debris. OA serum and synovial fluid samples were obtained from patients diagnosed with knee OA (of Kellgren-Lawrence score 2 to 4 [17]) according to the 1985 criteria of the American Rheumatism Association [18]. For mass spectrometric analysis, OA synovial fluid samples were from five Caucasian men aged 50 to 75 years who met the 1985 OA criteria [18]; exclusion criteria included radiographic evidence of chondrocalcinosis or evidence of crystals under polarizing microscopy. Demographics and clinical characteristics of these five individuals are shown in Table 1. Synovial fluids from the other OA patients and from the RA patients were provided as de-identified remnant clinical samples, and patient demographics were therefore unavailable for these samples. All RA patients met the 1987 American Rheumatism Association criteria for RA [19] and had RA of less than 6 months' duration; exclusion criteria included concurrent infectious or crystal arthritis. Samples of "normal" serum were obtained from healthy individuals who had no joint pain and no radiographic evidence of knee arthritis [20]. OA and normal sera were matched by age, sex, and BMI. Serum and synovial fluid samples were not matched but were derived from patients with the characteristics described earlier. All samples were aliquoted and stored at -80°C.

**Table 1:** Clinical and demographic characteristics of OA patients whose synovial fluid was analyzed with mass spectrometry

Subject^a^	Age (years)	K-L score	SF cell count (cells/mm^3^)
1	43	3	1,090

2	70	3	400

3	68	2	850

4	72	4	Not measured

5	74	4	Not measured

K-L, Kellgren-Lawrence OA score; SF, synovial fluid. ^a^All subjects were male and Caucasian with symptomatic pain in the aspirated knee.

### Mass spectrometric analysis

Synovial fluid proteins were separated by 1D or 2D polyacrylamide gel electrophoresis (PAGE), trypsinized, and identified by liquid chromatography tandem mass spectrometry (LCMS), as follows. Fifty microliters of frozen synovial fluid was diluted to a final volume of 1 ml in phosphate buffered saline (PBS) containing Halt protease and phosphatase inhibitor (Thermo Fisher Scientific), and then depleted of the highly abundant proteins albumin and immunoglobulin G (IgG) by using the ProteoPrep Immunoaffinity Albumin & IgG Depletion Kit (Sigma-Aldrich) according to the manufacturer's instructions. In brief, synovial fluids were twice passed over spin columns prepacked with a mixture of two beaded mediums containing recombinantly expressed, small, single-chain antibody ligands. The flow-through fractions containing synovial fluid depleted of albumin and IgG were diluted 1:1 with Laemmli Sample Buffer (BioRad) and then subjected to 1D-PAGE or 2D-PAGE analysis. Because a small number of proteins other than albumin and IgG may bind to the medium in the spin columns, the bound proteins were eluted with Laemmli sample buffer and also subjected to PAGE analysis. For 1D-PAGE analysis, proteins were boiled for 10 minutes and separated on Precast Criterion XCT gels (4% to 12% linear gradient, BioRad). After electrophoresis, the gels were stained for 1 hour with Gelcode blue (Pierce) and destained overnight. For 2D-PAGE analysis, methods were as previously described [21]. In brief, 100 μg of synovial fluid proteins was dissolved in 150 μl of isoelectric focusing (IEF) buffer (ReadyPrep Sequential Extraction Kit Reagent 3, BioRad). For the first-dimension electrophoresis, 150 μl (at a 1 μg/μl concentration) of sample solution was applied to an 11-cm Ready-Strip Immobilized pH Gradient (IPG) strip, pH 3 to 10 (BioRad). The IPG strips were soaked in the sample solution for 1 hour, to allow uptake of the proteins, and then actively rehydrated in the Protean IEF cell (BioRad) for 12 hours at 50 V. IEF was performed for 1 hour at each of 100, 200, 500, and 1,000 V, and then for 10 hours at 8,000 V. For second-dimension electrophoresis, IPG strips were equilibrated for 20 minutes in 50 m*M *Tris-HCl, pH 8.8, containing 6 *M *urea, 1%SDS, 30% glycerol, and 65 m*M *dithiothreitol (DTT), and then re-equilibrated for 20 minutes in the same buffer containing 260 m*M *iodacetamide in place of DTT. Precast Criterion XCT gels (4% to 12% linear gradient, BioRad) were used for the second-dimension electrophoresis, as was done for the 1D-PAGE. After electrophoresis, the gels were stained for 1 hour with Gelcode blue (Pierce) and destained overnight.

The stained protein bands and spots (from the 1D-PAGE and 2D-PAGE, respectively) were cut out of the gels, immersed in 10 m*M *ammonium bicarbonate containing 10 m*M *DTT and 100 m*M *iodoacetamide, treated with 100% acetonitrile, and then digested overnight at 37°C with 0.1 mg trypsin (Sigma-Aldrich) in 10 m*M *ammonium acetate containing 10% acetonitrile. The trypsinized proteins were identified with LCMS by using the Agilent 1100 LC system and the Agilent XCT Ultra Ion Trap (Agilent Technologies, Santa Clara, CA) as previously described [22]. We scanned the LCMS data against the SwissProt database by using the SpectrumMill software (Agilent). We required the detection of at least two peptides for identification of a protein, and a significance level of *P *≤ 0.05 for identification of each peptide. The significance level of peptide identification takes into account the number of ionization forms of the fragmented peptide that match with a particular protein in the SwissProt database (with penalties for ionization forms not identified), as well as the total intensity of each ionization form [23].

### Multiplex cytokine analysis

Multiplex analysis of cytokines and chemokines in human serum and synovial fluid samples was performed by using both the 27-plex and the 21-plex Bio-Plex Pro Human Cytokine Assay (BioRad) run on the Luminex 200 platform, as recommended by the manufacturers. Performing the Bio-Plex assay with the kit reagents, we found that several commercial reagents designed to block the confounding effect of heterophilic antibodies, including ones we used previously with other cytokine assay kits [24], did not significantly affect the readout of the Bio-Plex assay; we therefore did not use such blocking reagents with the Bio-Plex assay. Data processing was performed by using Bio-Plex Manager 5.0, and analyte concentrations (in picograms per milliliter) were interpolated from standard curves. Statistical differences in cytokine levels were calculated with significance analysis of microarrays (SAM [25]), and the SAM-generated results with a false discovery rate (FDR) of less than 10% were selected. To identify relations and to display our results most effectively, we normalized the analyte concentrations as follows: all values less than 1 were designated as 1, and the mean concentration of each analyte in the "normal serum" samples was calculated; the analyte value in the sample was then divided by the mean analyte value in normal serum, and finally, a log-base-2 transformation was applied. Results were subjected to unsupervised hierarchic clustering by using Cluster 3.0, which arranges the SAM-generated results according to similarities in cytokine levels, and the clustering results were displayed by using Java Treeview (Version 1.1.3).

### Macrophage stimulation assays

To generate mouse macrophages, we differentiated bone-marrow cells isolated from wild-type C57BL/6 mice and from B6.B10ScN-*Tlr4^lps-del ^*mice (Jackson Laboratory) according to standard procedures [26]. In brief, the femur and tibia were flushed with α-minimal essential medium (MEM; Invitrogen) by using a 1-ml syringe and a 25-gauge needle. The resulting cell suspension was lysed with ACK Lysing Buffer (Invitrogen) for removal of erythrocytes. Cell clumps were removed by filtering through a 70-μm cell strainer (BD). The remaining cells in the suspension were cultured on 100-mm culture dishes in α-MEM supplemented with 10% fetal bovine serum (FBS), 100 units/ml of penicillin, 100 μg/ml of streptomycin, and 2 m*M *glutamine (Invitrogen) for 16 to 24 hours in 5% CO_2 _at 37°C. Nonadherent cells were collected, plated on 100-mm dishes, and differentiated into bone-marrow-derived macrophages (BMMs) for 6 days in the presence of 30 ng/ml of macrophage colony-stimulating factor (PeproTech). To generate human monocyte-derived macrophages (MDMs), we collected peripheral blood mononuclear cells (PBMCs) by performing density-gradient centrifugation of LRS chamber content (Stanford Blood Center) over Ficoll (Invitrogen), purified human monocytes by negative selection by using a monocyte isolation kit (Miltenyi Biotec), and differentiated the monocytes into macrophages by culturing them for 7 days in RPMI containing 10% FBS and 30 ng/ml of human M-CSF.

For stimulation assays, mouse BMMs were plated in 96-well plates at 1 × 10^5 ^cells/well, and human macrophages at 7 × 10^4 ^cells/well. Cells were incubated for 24 hours with lipopolysaccharide (LPS; Sigma-Aldrich), peptidoglycan (InvivoGen), α_1_-microglobulin (Cell Sciences), α2-macroglobulin (EMD Chemicals), α1-acid glycoprotein (EMD Chemicals), Gc-globulin (also known as vitamin D-binding protein; Abcam), haptoglobin (Sigma-Aldrich), or human serum albumin (Sigma-Aldrich). We measured levels of interleukin-1β (IL-1β), interleukin-6 (IL-6), and vascular endothelial growth factor (VEGF) in the culture supernatants with Luminex analysis, by using a 27-plex Bio-Plex Pro Human Cytokine Assay kit (BioRad) according to the manufacturer's instructions. We measured TNF levels with enzyme-linked immunosorbent assay (ELISA; PeproTech). For the TNF ELISA, the limits of detection were 16 to 2,000 pg/ml for mouse TNF, and 23 to 1,500 pg/ml for human TNF. For the Luminex assay, the limits of detection were 3.2 to 3,261 pg/ml for IL-1β, 2.3 to 18,880 pg/ml for IL-6, and 5.5 to 56,237 pg/ml for VEGF. To exclude a contribution of endotoxin contamination, we included 10 μg/ml of polymyxin B (Sigma-Aldrich) in some of the stimulation assays. As an additional control for endotoxin contamination, we tested whether preincubating the plasma proteins with proteinase K and β-mercaptoethanol at 55°C for 4 hours (and then at 100°C for 10 minutes to inactivate the proteinase K) abrogated their ability to induce the production of cytokines (the plasma proteins, but not any contaminating endotoxin, would be denatured under these conditions).

### Statistical analysis

One-way ANOVA and unpaired *t *test (Graph-Pad Software) were used to analyze differences in levels of cytokines. *P *values less than 0.05 were considered significant.

## Results and Discussion

We first used mass spectrometry to survey the proteins present in the synovial fluid of patients with knee OA. Synovial fluid proteins from five OA patients were separated by 1D- or 2D-PAGE and then identified by LCMS. Analysis of all five samples identified a total of 111 unique proteins; three of these were keratin proteins, skin proteins most likely obtained as a result of the cutaneous puncture performed during aspiration of the synovial joints. Eliminating these keratins left 108 unique proteins (Tables 2 and 3), most of which were detected in all synovial fluid samples analyzed. Of these, 44 were identified in a previous proteomic survey of highly abundant proteins in OA synovial fluid [10] (Table 2). Thus, we confirmed the presence of serine protease inhibitors (for example, antithrombin III, α_1_-antitrypsin, α_1_-antichymotrypsin, kininogen 1) and of proteins important in regulating proteases that degrade cartilage ECM. We also confirmed the presence of proteins involved in cartilage (for example, fibronectin) and/or collagen (for example, gelsolin and collagen α_1_, α_2_, and α_3 _chains) metabolism, and of proteins involved in inflammation or immunity (for example, fibrinogen, AGP 1, complement factors, immunoglobulins, cytokines) (Table 2), findings consistent with the inflammation, ECM degradation, and immune-cell infiltration that characterize OA. Among the 64 proteins that we newly identified (Table 3) were histone-related proteins, macrophage-related proteins, proinflammatory receptors, and proteins related to the proinflammatory transcription factor nuclear factor kappa B (Table 4), presumably reflecting the turnover of resident synovial cells or infiltrating inflammatory cells.

**Table 2:** Proteins identified in OA synovial fluids in this study and in a previous proteomic study [10]

Protein name^a^	Accession no.^b^	Score^c^	% Coverage^d^	Number of peptides^e^
**Transferrin**	P02787	877.49	65	57

**α_2_-Macroglobulin**	P01023	569.94	34	57

**Serum albumin**	P02768	514.49	60	34

**Complement C3**	P01024	426.92	27	34

**Apolipoprotein A-I**	P02647	405.32	67	26

**α_1_-Antitrypsin (α_1 _protease inhibitor)**	P01009	388.37	66	27

**Apolipoprotein A-IV**	P06727	370.16	60	27

**Haptoglobin**	P00738	331.09	49	22

Hemopexin (β-1B-glycoprotein)	P02790	273.36	40	20

**Gc-globulin (vitamin D-binding protein precursor)**	P02774	269.18	43	21

**α_1_B-glycoprotein**	P04217	215.74	47	16

**Complement factor B (C3/C5 convertase)**	P00751	163.54	20	12

**α_1_-Antichymotrypsin**	P01011	144.7	27	10

**Antithrombin-III**	P01008	142.4	32	10

**Ceruloplasmin (EC 1.16.3.1) (Ferroxidase)**	P00450	132.65	12	9

**Transthyretin**	P02766	129.52	69	8

**Plasma protease C1 inhibitor**	P05155	111.87	17	8

Ig mu chain C region	P01871	98.51	16	7

Actin, cytoplasmic 2 (γ-actin)	P63261	89.13	33	8

**Fibrinogen β chain**	P02675	88.28	18	7

**α_2_-HS-glycoprotein**	P02765	85.26	22	7

**Complement factor H (H factor 1)**	P08603	80.47	8	8

**α_1_-Acid glycoprotein 1 (Orosomucoid-1)**	P02763	68.98	23	5

**Complement C5**	P01031	55.88	8	7

**Prothrombin (coagulation factor II)**	P00734	52.57	9	4

**Plasma retinol-binding protein**	P02753	50.79	20	4

**Apolipoprotein E**	P02649	49.32	21	4

**Afamin**	P43652	48.67	7	4

**Fibrinogen α chain**	P02671	48.63	6	4

Gelsolin	P06396	47.03	4	3

**Complement C4**	P01028	39.34	8	6

Vitronectin	P04004	38.1	6	3

**Apolipoprotein A-II**	P02652	36.26	22	3

Hemoglobin β subunit (Hemoglobin β chain)	P68871	31.88	17	2

**β_2_-Glycoprotein I (apolipoprotein H)**	P02749	30.33	6	2

Inter-α-trypsin inhibitor heavy chain H4	Q14624	28.43	5	3

**Fibronectin**	P02751	23.84	2	2

**Clusterin**	P10909	21.17	12	2

**Complement component C8 γ chain**	P07360	20.93	10	2

Histidine-rich glycoprotein	P04196	20.11	5	2

**Fibrinogen γ chain**	P02679	19.9	7	2

**Kininogen-1**	P01042	16.02	4	2

Desmoplakin	P15924	11.59	1	2

**α_1_-Microglobulin/bikunin precursor protein precursor**	P02760	11.13	9	2

^a^Shown in bold are the proteins classified as plasma proteins (as assessed by www.HPRD.org). ^b^UniProtKB/Swiss-Prot database. ^c^An aggregate score of the quality of the peptide spectra obtained, the total number of peptides identified, and percentage coverage of the protein. ^d^The number of amino acids identified as a percentage of the total number of amino acids in the corresponding protein. ^e^The number of peptides identified that correspond to the protein indicated.

**Table 3:** Proteins newly identified in OA synovial fluids

Protein name^a^	Accession no.^b^	Score^c^	% Coverage^d^	No. of peptides^e^
Zinc-α_2_-glycoprotein	P25311	140.54	35	10

Ig α1 chain C region	P01876	128.82	37	9

Ig γ-1 chain C region	P01857	115.3	35	9

Ig κ chain C region	P01834	87.06	85	6

Calgranulin A (MRP-8)	P05109	67.87	33	5

Collagen α_1_(I) chain	P02452	40.14	4	4

**Complement component C9**	P02748	36.76	9	4

**Serum paraoxonase/arylesterase 1**	P27169	34.77	13	3

Ig κ chain V-III region SIE	P01620	33.65	38	3

Ig heavy-chain V-III region BRO	P01766	28.94	24	2

NF-κB-repressing factor	O15226	23.76	8	3

A-kinase anchor protein-like protein 8	Q9ULX6	23.65	11	3

Structural maintenance of chromosomes 4-like 1 protein	Q9NTJ3	23.43	1	2

Bromodomain adjacent to zinc-finger domain 2B (hWALp4)	Q9UIF8	22.6	3	3

Histone deacetylase 5	Q9UQL6	22.4	4	3

Voltage-dependent R-type calcium channel α-1E subunit	Q15878	22.29	1	2

Inhibitor of nuclear factor κB kinase β subunit	O14920	22.07	8	3

β platelet-derived growth factor receptor precursor (EC 2.7.1.112) (CD140b antigen)	P09619	20.93	5	2

Leucine-rich α_2_-glycoprotein	P02750	20.55	5	2

Mitochondrial 28S ribosomal protein S29	P51398	19.76	6	2

Glucose-6-phosphate 1-dehydrogenase	P11413	19.58	14	3

Collagen α_2_(I) chain	P08123	18.98	7	3

Histone H4	P62805	18.73	16	2

Macrophage inflammatory protein 2-β	P19876	18.72	28	2

Collagen α_3_(IV) chain	Q01955	18.15	5	2

Histone-lysine *N*-methyltransferase, H3 lysine-9 specific 4	Q15047	17.82	4	2

Toll-like receptor 6	Q9Y2C9	17.38	2	2

Nuclear factor NF-κB p100 subunit	Q00653	17.22	7	2

Possible global transcription activator SNF2L1 (SWI/SNF-related matrix-associated actin-dependent regulator of chromatin subfamily A member 1)	P28370	16.37	5	2

RRP5 protein homologue	Q14690	16.35	3	2

Interleukin-20 receptor α chain	Q9UHF4	16.22	11	2

NF-κB inhibitor-like protein 1	Q9UBC1	16.13	14	2

Platelet endothelial cell-adhesion molecule	P16284	15.87	10	2

Fibroblast growth factor receptor 2	P21802	15.4	2	2

Interleukin-1 receptor-associated kinase 1	P51617	14.92	11	2

Cadherin EGF LAG seven-pass G-type receptor 1	Q9NYQ6	14.84	1	2

Collagen α_2_(V) chain	P05997	14.78	5	2

Zinc-finger A20 domain-containing protein 1	Q6GQQ9	14.61	6	2

Low-density lipoprotein receptor-related protein 1 (apolipoprotein E receptor)	Q07954	14.54	1	2

Microtubule-actin crosslinking factor 1, isoforms 1/2/3/5	Q9UPN3	14.53	1	2

Zinc-finger DHHC domain-containing protein 13	Q8IUH4	14.48	11	2

Death-associated protein kinase 1	P53355	14.41	3	2

Collagen α_1_(V) chain	P20908	13.78	4	2

Integrin α-V (Vitronectin receptor α subunit)	P06756	13.52	3	2

Misshapen-like kinase 1(MAPK/ERK kinase kinase kinase 6)	Q8N4C8	13.36	4	2

Cadherin EGF LAG seven-pass G-type receptor	Q9NYQ7	13.26	1	3

Regulator of G-protein signaling 14	O43566	13.21	8	2

Collagen α_1_(VII) chain	Q02388	13.19	1	2

Interleukin-12 receptor β_2 _chain	Q99665	13.1	5	2

Interleukin-1β (IL-1β)	P01584	12.94	20	2

Platelet-derived growth factor B chain	P01127	12.91	18	2

Collagen α_1_(II) chain	P02458	12.65	6	2

Pappalysin-1	Q13219	12.58	2	2

**Complement C1q tumor necrosis factor-related protein 5**	Q9BXJ0	11.97	23	2

Interleukin-18 receptor 1 precursor (IL-1 receptor-related protein)	Q13478	11.8	4	2

A kinase anchor protein 10, mitochondrial (PRKA10)	O43572	11.36	8	2

Neutrophil collagenase	P22894	11.34	9	2

Protein-arginine deiminase type I	Q9ULC6	11.34	5	2

TNF receptor-associated factor 5 (RING finger protein 84)	O00463	10.92	8	2

Macrophage receptor MARCO	Q9UEW3	10.89	8	2

Collagen α_3_(V) chain	P25940	10.32	1	2

Histone acetyltransferase MYST3	Q92794	10.14	2	2

Leukocyte immunoglobulin-like receptor subfamily B member 4	Q8NHJ6	9.43	11	2

Mast/stem cell growth-factor receptor	P10721	9.3	5	2

^a^Shown in bold are the proteins classified as plasma proteins (as assessed by www.HPRD.org). ^b^UniProtKB/Swiss-Prot database. ^c^An aggregate score of the quality of the peptide spectra obtained, the total number of peptides identified, and percentage coverage of the protein. ^d^The number of amino acids identified as a percentage of the total number of amino acids in the corresponding protein. ^e^The number of peptides identified that correspond to the protein indicated.

**Table 4:** Classes of proteins newly identified in OA synovial fluids

Histone-related proteins	NF-κB-related proteins	Inflammatory receptors	Macrophage-related proteins
Histone deacetylase 5	NF-κ-B-repressing factor	IL-12-receptor β_2_	Macrophage inflammatory protein 2-β

Histone H4	Inhibitor of nuclear factor κB kinase β subunit	IL-18-receptor 1	Macrophage receptor MARCO

Histone-lysine *N*- methyltransferase, H3 lysine-9 specific 4	Nuclear factor κB p100 subunit	IL-20-receptor α chain	

Histone acetyltransferase MYST3	NF-κB inhibitor-like protein 1	Toll-like receptor 6	

		Integrin-αV	

**Figure 1:**
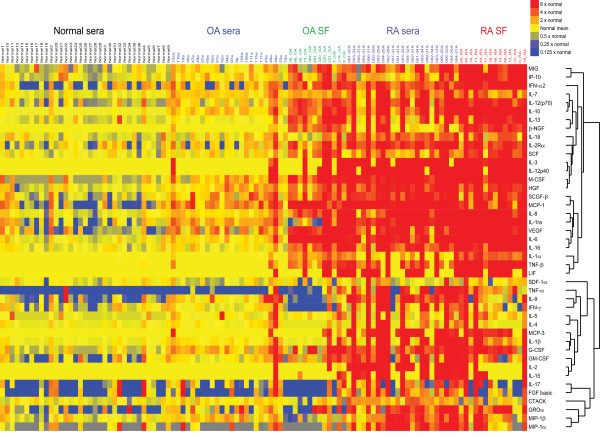
**Inflammatory cytokines are associated with osteoarthritis**. Relative cytokine levels in serum and synovial fluid (SF) samples from patients with osteoarthritis (OA) or rheumatoid arthritis (RA) and in serum samples from healthy individuals (normal sera). Cytokine levels were measured with a multiplex bead-based immunoassay. Samples from individual patients are listed above the heatmap, and the individual cytokines are listed to the right of the heatmap. IL, interleukin; IFN, interferon; MIG, monokine induced by IFN-γ; IP-10, interferon gamma-induced protein 10; IL-1ra, interleukin-1 receptor antagonist; VEGF, vascular endothelial growth factor; GM-CSF, granulocyte macrophage colony-stimulating factor; FGF, fibroblast growth factor; MCP, monocyte chemotactic protein; IL-2Rα, interleukin-2 receptor α chain; HGF, hepatocyte growth factor; GROα, growth-regulated oncogene α; MIP-1, macrophage inflammatory protein; β-NGF, β nerve growth factor; SCF, stem cell factor; M-CSF, macrophage colony-stimulating factor; SCGF-β, stem cell growth factor β; LIF, leukemia inhibitory factor; SDF-1α, stromal cell-derived factor 1α; G-CSF, granulocyte colony-stimulating factor; CTACK, cutaneous T-cell attracting chemokine.

**Figure 2:**
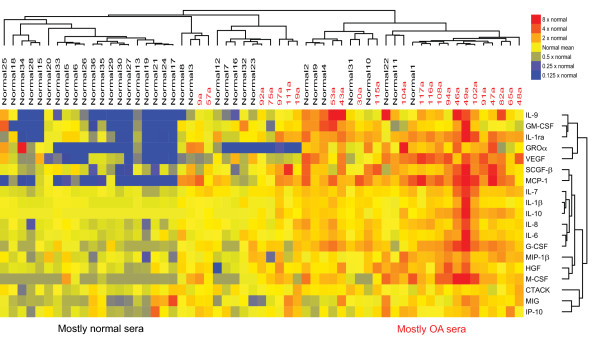
**Levels of inflammatory cytokines are higher in OA compared with healthy sera**. Cytokines whose levels differ significantly between sera from individuals with osteoarthritis (OA) and sera from age-matched healthy individuals (FDR < 10%). Significance analysis of microarrays (SAM) was used to identify statistically significant differences, and the SAM-generated results were subjected to unsupervised hierarchic clustering. Cytokine levels were measured with a multiplex bead-based immunoassay. Samples from individual patients are listed above the heatmap, and the individual cytokines are listed to the right of the heatmap. IL, interleukin; MIG, monokine induced by IFN-γ; IP-10, interferon gamma-induced protein 10; IL-1ra, interleukin-1 receptor antagonist; VEGF, vascular endothelial growth factor; GM-CSF, granulocyte macrophage colony-stimulating factor; MCP, monocyte chemotactic protein; HGF, hepatocyte growth factor; GROα, growth-regulated oncogene α; MIP-1β, macrophage inflammatory protein 1β; M-CSF, macrophage colony-stimulating factor; SCGF-β, stem cell growth factor β; G-CSF, granulocyte colony-stimulating factor; CTACK, cutaneous T-cell attracting chemokine.

**Figure 3:**
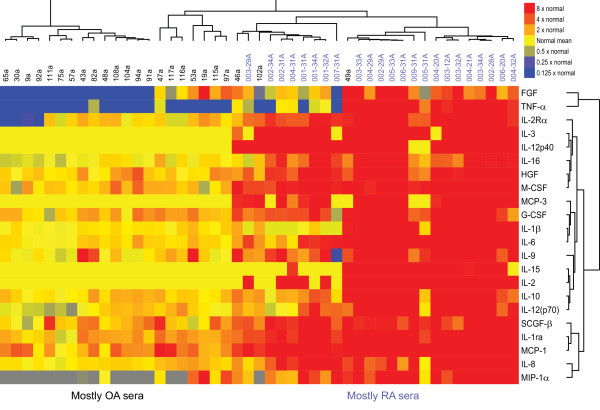
**Levels of inflammatory cytokines are higher in RA compared with OA sera**. Cytokines whose levels differ significantly between sera from individuals with osteoarthritis (OA) and sera from individuals with rheumatoid arthritis (RA) (FDR < 10%). Significance analysis of microarrays (SAMs) was used to identify statistically significant differences, and the SAM-generated results were subjected to unsupervised hierarchic clustering. Cytokine levels were measured with a multiplex bead-based immunoassay. Samples from individual patients are listed above the heatmap, and the individual cytokines are listed to the right of the heatmap. IL, interleukin; IL-1ra, interleukin-1 receptor antagonist; FGF, fibroblast growth factor; MCP, monocyte chemotactic protein; HGF, hepatocyte growth factor; MIP-1, macrophage inflammatory protein; M-CSF, macrophage colony-stimulating factor; SCGF-β, stem cell growth factor β; G-CSF, granulocyte colony-stimulating factor.

Our mass-spectrometric findings revealed the presence of many molecules associated with inflammation. Although cytokines are also classically associated with inflammation, PAGE-based mass spectrometry is not well suited to the detection of small proteins such as cytokines. We therefore used a multiplex immunoassay to measure levels of inflammatory cytokines and chemokines in synovial fluid samples from 12 patients with knee OA and 14 patients with RA, as well as in serum samples from 24 patients with knee OA, 23 patients with RA, and 35 healthy individuals. Samples from patients with RA, a classic inflammatory arthritis, were used as a comparator. Figure 1 shows a heatmap of the relative levels of cytokines in the five groups of samples. Compared with cytokine levels in normal sera, cytokine levels in OA sera were generally slightly higher, and those in RA sera were much higher (Figure 1). SAM analysis revealed that levels of several inflammatory cytokines (for example, IL-1β and IL-6), chemokines (for example, IP-10 (also known as CXCL10), MCP-1, IL-8, MIG, and MIP-1β), and growth factors (for example, VEGF and SCGF-β) were significantly higher in OA sera than in normal sera (FDR < 10%; Figure 2), consistent with previous reports of the association of OA with such inflammatory mediators [27]. Interestingly, we also found OA-associated elevations in levels of IL-9 and cutaneous T-cell attracting chemokine (CTACK), supporting the concept that T cells play a role in OA [28]. As expected, cytokine levels were significantly higher in RA sera than in OA sera (Figure 3; FDR < 10%).

Unlike RA, OA is considered a disorder that is restricted to the joints. Indeed, levels of multiple cytokines were much higher in OA synovial fluids than in OA sera (Figure 1 and Table 5); levels of TNF were negligible in OA sera but substantial in OA synovial fluid (Figure 1 and Table 5). Our findings suggest that the abnormally high levels of cytokines in OA sera largely reflect overproduction of these cytokines in the joint, consistent with the finding that levels of high-sensitivity C-reactive protein in the serum of OA patients correlate with the degree of inflammatory infiltrate in the patients' joints [29]. Thus, OA is associated with low-grade inflammation that may originate in the joints.

**Table 5:** Absolute and relative cytokine levels in healthy and OA serum, and in OA synovial fluid

Cytokine	Normal serum levels (pg/ml)^a^	OA serum levels (pg/ml)^b^	OA SF levels (pg/ml)^c^	Ratio of OA serum levels to normal serum levels	Ratio of OA SF levels to OA serum levels
IL-6	3.02 (2.7-4.4)	5.13 (4.4-5.8)	975.39 (454.0-2,689.5)	1.7	190.1

IL-1β	1.22 (1.0-1.3)	1.58 (1.4-1.8)	1.14 (1.0-1.7)	1.3	0.7

TNF	0.00 (0.0-0.0)	0.00 (0.0-0.0)	2.92 (0.0-13.3)	-	-

VEGF	20.53 (11.7-65.6)	78.22 (31.3-124.6)	496.31 (245.0-577.8)	3.8	6.3

MCP-1	3.59 (0.0-11.9)	18.53 (13.0-28.0)	107.67 (84.8-191.1)	5.2	5.8

IP-10	537.00 (376.1-750.4)	795.55 (684.7-1,029.7)	2,105.40 (923.2-4,913.3)	1.5	2.6

MIG	244.51 (173.5-374.6)	420.13 (308.4-568.6)	1,047.14 (389.8-1,925.3)	1.7	2.5

OA, osteoarthritis; SF, synovial fluid; IL, interleukin; TNF, tumor necrosis factor; VEGF, vascular endothelial growth factor; MCP-1, macrophage chemotactic protein 1; IP-10, interferon γ-induced protein 10; MIG, monokine-induced by interferon γ. ^a^The median (IQR); *n *= 34 healthy individuals. ^b^The median (IQR); *n *= 23 individuals with OA. ^c^The median (IQR); *n *= 10 individuals with OA.

Interestingly, 39 (36%) of the proteins we identified in OA synovial fluid are classically considered plasma proteins (Tables 2 and 3). Indeed, plasma proteins form a large proportion of the proteins enriched in OA synovial fluid relative to healthy synovial fluid [10]. What might these plasma proteins be doing in the OA joint? Like certain products of ECM breakdown [2,5,6], the plasma protein fibrinogen can function as a DAMP and has been proposed to contribute to the pathogenesis of inflammatory arthritis [7-9]. We therefore examined whether other plasma proteins in OA synovial fluid can function as immunostimulatory DAMPs that could contribute to the low-grade inflammation associated with OA.

Key players in OA-associated inflammation are the macrophages [27,30,31]. The cell infiltrate in human OA joints consists mainly of macrophages, and mice depleted of macrophages are relatively resistant to collagenase-induced OA [30]. Macrophages from OA joints produce a number of growth factors, such as VEGF, and inflammatory cytokines, such as the major OA-associated cytokines IL-1β and TNF [30]. We detected VEGF, IL-1β, and TNF in OA synovial fluid in our cytokine screen (Figure 1 and Table 5) and found that levels of VEGF and IL-1β are significantly higher in OA sera than in normal sera (Figure 2). VEGF may promote OA pathology by inducing angiogenesis (and thereby osteophyte formation) and by inducing matrix metalloprotease production (and thereby cartilage degradation) [32]. The cytokines produced by macrophages amplify the inflammation in the joints by inducing synovial cells to produce further cytokines and chemokines, as well as matrix metalloproteases [30]. Moreover, macrophages express many of the receptors that mediate DAMP signaling, and they can thus trigger an inflammatory cascade in response to DAMPs present in OA synovial fluid [7-9].

**Figure 4:**
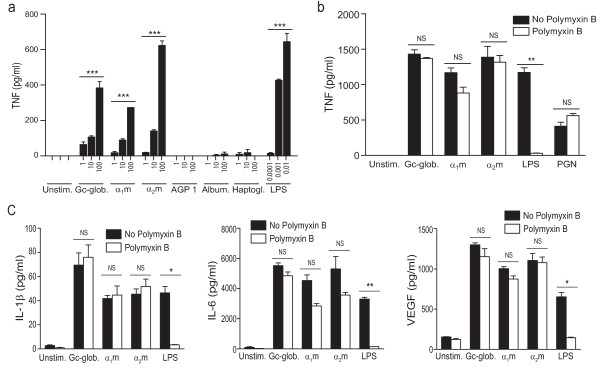
**Plasma proteins detected in osteoarthritic synovial fluid are immunostimulatory**. Mouse bone-marrow-derived macrophages (BMMs) and human monocyte-derived macrophages (MDMs) were stimulated for 24 hours with plasma proteins detected in osteoarthritic synovial fluid (Table 2), after which cytokine levels in the supernatants were measured with ELISA or Luminex immunoassay. **(a) **Levels of TNF produced by mouse BMMs stimulated with the indicated concentrations (in μg/ml) of Gc-globulin, α_1_-microglobulin (α_1_m), α_2_-macroglobulin (α_2_m), α_1_-acid glycoprotein 1 (AGP 1), albumin, or haptoglobin. Lipopolysaccharide (LPS) was used as a positive control. **(b) **Levels of TNF produced by human MDMs stimulated with 50 μg/ml of α_1_m, α_2_m, or Gc-globulin in the presence or absence of 10 μg/ml of polymyxin B, an inhibitor of LPS. LPS (1 ng/ml) was used as a positive control for the efficacy of polymyxin B, and peptidoglycan (PGN; 5 μg/ml), as a negative control. **(c) **Levels of interleukin-1β (IL-1β), interleukin-6 (IL-6), and vascular endothelial growth factor (VEGF) produced by human MDMs stimulated with 50 μg/ml of Gc-globulin, α_1_m, or α_2_m. LPS (1 ng/ml) was used as a positive control. Results are representative of experiments performed at least twice. In **(a)**, data are shown as the mean ± SEM of duplicates. In **(b) **and **(c)**, data are shown as the mean ± SEM of triplicates. *P < 0.05; **P < 0.01, ***P < 0.001; NS, not significant.

We therefore assessed whether a subset of the identified plasma proteins could induce macrophages to produce TNF, a key cytokine that is thought to drive the inflammatory cascade in OA [27]. We tested α_1_-microglobulin (α_1_m), α_1_-acid glycoprotein 1 (AGP 1; also known as orosomucoid 1), α_2_-macroglobulin (α_2_m), Gc-globulin (also known as vitamin D-binding protein), albumin, and haptoglobin, all of them plasma proteins detected in our survey of synovial fluid proteins (Table 2) and shown to be enriched in OA synovial fluid [10]. With mouse macrophages, we found that α_1_m, α_2_m, and Gc-globulin, at concentrations similar to those measured in synovial fluid [33-35], each dose-dependently stimulated the production of TNF, whereas AGP 1, albumin, and haptoglobin did not (Figure 4a). The plasma proteins ceruloplasmin, complement component C3, complement component C4, β_2_-glycoprotein (also known as apolipoprotein H) also did not stimulate TNF production (data not shown).

**Figure 5:**
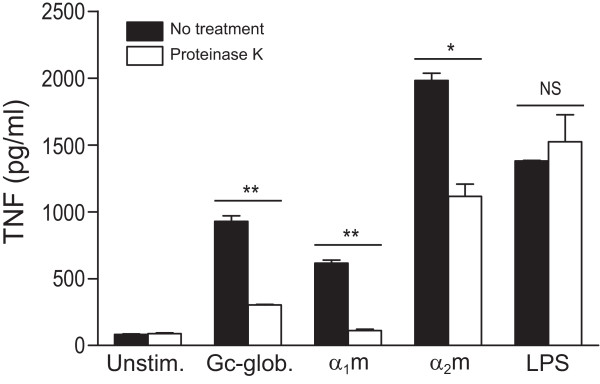
**Induction of TNF production by plasma proteins is not due to endotoxin contamination**. RAW264.7 macrophages were stimulated for 24 hours with 50 μg/ml of Gc-globulin, α_1_-microglobulin (α_1_m), or α_2_-macroglobulin (α_2_m) that had been incubated with proteinase K (20 μg/ml) at 55°C for 4 hours in the presence of β-mercaptoethanol and then heated to 100°C for 10 minutes. TNF levels in the supernatants were determined with ELISA. Lipopolysaccharide (LPS; 1 ng/ml) was used as a positive control. Data are shown as the mean ± SEM of duplicates from one of two representative experiments. *P < 0.05; **P < 0.01.

We next examined the effect of α_1_m, α_2_m, and Gc-globulin on cytokine production in human macrophages. Because the endotoxin LPS is a common contaminant and is itself an agonist of TLR4, we tested the stimulatory properties of the plasma proteins in the presence of polymyxin B, a compound that neutralizes LPS. In the presence of polymyxin B, α_1_m-, α_2_m-, and Gc-globulin-induced TNF production was not significantly reduced, whereas LPS-induced TNF production was abrogated (Figure 4b). Additionally, pretreatment with proteinase K significantly abrogated TNF production induced by the plasma proteins but not TNF production induced by LPS (Figure 5). Although we cannot exclude the possibility that a small component of the observed stimulation is due to endotoxin, this result confirms that the plasma proteins are themselves immunostimulatory. Gc-globulin, α_1_m, and α_2_m were also able to induce the production of several other inflammatory cytokines that were upregulated in OA serum and synovial fluid (Figures 1 and 2): IL-1β, IL-6, and VEGF (Figure 4c). Thus, Gc-globulin, α_1_m, and α_2_m can each induce the production of TNF, IL-1β, IL-6, and VEGF, all molecules implicated in the pathogenesis of OA [27,30,31].

**Figure 6:**
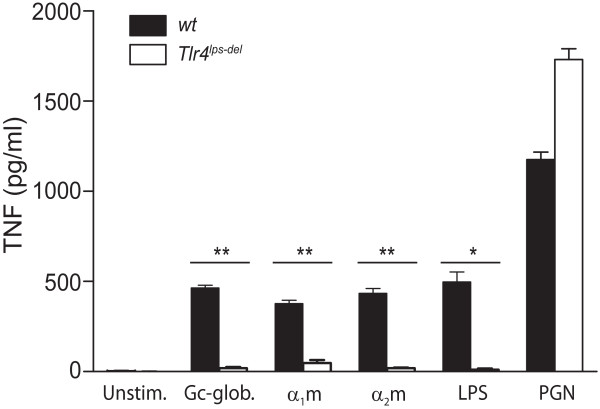
**Plasma proteins detected in osteoarthritic synovial fluid stimulate macrophage TNF production via TLR4**. Levels of TNF produced by wild-type (*wt*) or TLR4-deficient (*Tlr4^lps-del^*) mouse bone-marrow-derived macrophages stimulated for 24 hours with 50 μg/ml of Gc-globulin, α_1_-microglobulin (α_1_m), or α_2_-macroglobulin (α_2_m), after which TNF levels in the supernatants were determined with ELISA. Lipopolysaccharide (LPS; 1 ng/ml) was used as a positive control for TLR4-dependent TNF production, and peptidoglycan (PGN; 5 μg/ml) as a positive control for TLR4-independent TNF production. Data are shown as the mean ± SEM of triplicates from one of three representative experiments. *P < 0.05; **P < 0.01.

But how do these plasma proteins stimulate cytokine production? To determine whether these immunostimulatory plasma proteins signal through TLR4, we examined whether Gc-globulin, α_1_m, and α_2_m could also induce TNF production in TLR4-deficient macrophages. TLR4 deficiency inhibited Gc-globulin-, α_1_m-, and α_2_m-induced TNF production (Figure 6). Confirming that the defect in inflammatory signaling in the *Tlr4^lps-del ^*macrophages was specific to the TLR4 pathway, theTLR2-specific agonist peptidoglycan was able to induce TNF production in these cells-in fact, to a greater degree than in wild-type cells (possibly because of compensatory mechanisms operating within the TLR family) (Figure 6). Thus, Gc-globulin-, α_1_m-, and α_2_m-induced production of TNF is dependent on TLR4.

Interest in the putative immunomodulatory effects of α_1_m, α_2_m, and Gc-globulin is increasing, with both proinflammatory and antiinflammatory properties suggested for each of them [36-38].

For example, α_1_m has been shown to bind to the surface of various inflammatory cells and to either stimulate or inhibit the activation of human lymphocytes [38]. The immunoregulatory role of α_1_m in health and disease is likely to be context dependent. Gc-globulin, however, appears to be primarily proinflammatory: it enhances the neutrophil- and monocyte-chemotactic activity of the anaphylatoxin C5a [36] and, in its sialic-acid-free form, activates macrophages [39]. Here, we uncover an additional mechanism by which these plasma proteins could promote inflammation. We speculate that exudation into extravascular spaces at sites of tissue damage and inflammation may render these plasma proteins inflammatory by bringing them into contact with TLR-expressing macrophages. Our finding that certain plasma proteins present in OA synovial fluid can induce macrophage production of inflammatory cytokines supports the model of local production of inflammatory mediators in the joints in OA.

## Conclusions

We identified 108 proteins in OA synovial fluid and showed that OA is associated with low-grade inflammation. We found that plasma proteins form a large proportion of the proteins present in OA synovial fluid and that certain of these plasma proteins can signal through TLR4 to induce the production of an array of inflammatory cytokines, including those upregulated in OA. Our findings suggest that plasma proteins present in OA synovial fluid, whether through exudation from the plasma or production by synovial tissues, could contribute to low-grade inflammation in OA by functioning as DAMPs.

## Abbreviations

α_1_m: α_1_-microglobulin; AGP 1: α_1_-acid glycoprotein 1; α_2_m: α_2_-macroglobulin; BMM: bone-marrow-derived macrophage; DAMP: damage-associated molecular pattern; ECM: extracellular matrix; IL-1β: interleukin-1β; IL-6: interleukin-6; IP-10: interferon gamma-induced protein 10; LCMS: chromatography tandem mass spectrometry; LPS: lipopolysaccharide; MCP-1: macrophage chemotactic protein-1; MDM: monocyte-derived macrophage; MEM: minimal essential medium; MIG: monokine induced by interferon-γ; MIP-1: macrophage inflammatory protein-1; OA: osteoarthritis; PAGE: polyacrylamide gel electrophoresis; RA: rheumatoid arthritis; TLR: Toll-like receptor; TNF: tumor necrosis factor; VEGF: vascular endothelial growth factor.

## Competing interests

The authors declare that they have no competing interests.

## Authors' contributions

JS and WHR conceived the studies. OS performed the mass spectrometric analysis of synovial fluid. PEC, LJL, and JCE performed the multiplex cytokine analysis. JCE, KAB, and TPA collected and provided OA sera. DHS performed the *in vitro *macrophage stimulation assays. JS, WHR, DHS, OS, TML, and IH analyzed the resulting datasets. TML and JS wrote and edited the manuscript with the input of WHR, OS, and DHS. All authors read and approved the final manuscript.
